# Hybrid metamaterial switching for manipulating chirality based on VO_2_ phase transition

**DOI:** 10.1038/srep23186

**Published:** 2016-03-22

**Authors:** T. T. Lv, Y. X. Li, H. F. Ma, Z. Zhu, Z. P. Li, C. Y. Guan, J. H. Shi, H. Zhang, T. J. Cui

**Affiliations:** 1 Key Laboratory of In-Fiber Integrated Optics of Ministry of Education, College of Science, Harbin Engineering University, Harbin 150001, China; 2 State Key Laboratory of Millimeter Waves, Southeast University, Nanjing 210096, China; 3 SZU-NUS Collaborative Innovation Centre for Optoelectronic Science & Technology, and Key Laboratory of Optoelectronic Devices and Systems of Ministry of Education and Guangdong Province, Shenzhen University, Shenzhen 518060, China; 4 School of Electronic Science, Northeast Petroleum University, Daqing 163318, China

## Abstract

Polarization manipulations of electromagnetic waves can be obtained by chiral and anisotropic metamaterials routinely, but the dynamic and high-efficiency modulations of chiral properties still remain challenging at the terahertz range. Here, we theoretically demonstrate a new scheme for realizing thermal-controlled chirality using a hybrid terahertz metamaterial with embedded vanadium dioxide (VO_2_) films. The phase transition of VO_2_ films in 90° twisted E-shaped resonators enables high-efficiency thermal modulation of linear polarization conversion. The asymmetric transmission of linearly polarized wave and circular dichroism simultaneously exhibit a pronounced switching effect dictated by temperature-controlled conductivity of VO_2_ inclusions. The proposed hybrid metamaterial design opens exciting possibilities to achieve dynamic modulation of terahertz waves and further develop tunable terahertz polarization devices.

Polarization provides a controllable degree of freedom in the field of light propagation and information processing. Polarization devices are crucial in many optical systems. As a fundamental phenomenon, optical activity, that is the ability to rotate the polarization state of light, has acquired great importance in many fields of science including physics, life-sciences and chemistry^1^. This effect usually occurs in naturally chiral structures that lack mirror symmetry such as DNA, sugar solution and bio-molecules. However, chiral effect in natural media is normally weak and the devices are bulky. Recently, the advent and deep investigations of metamaterials and plasmonics open a promising platform to control the polarization states of light^2^,^3^,^4^. The artificial chirality in metamaterials is much stronger than that in natural media, therefore the corresponding polarization devices are possibly ultrathin and miniaturized that are suitable for lab-on-chip integration. Much attention has been paid to the chiral metamaterials^5^,^6^,^7^,^8^,^9^,^10^,^11^,^12^, as significant candidates, for flexibly manipulating the polarization state.

Terahertz (THz) wave occupies a large portion of the electromagnetic spectrum between microwave and infrared frequencies. A variety of metamaterials have been proposed to offer a strong engineered THz response and fill the so-called THz gap^13^,^14^,^15^,^16^,^17^,^18^,^19^,^20^,^21^,^22^. Many interesting phenomena have been studied in the THz regime, for instance, artificial magnetism^13^, negative refractive index^14^, anomalous refraction^15^, giant optical activity^16^,^17^ and asymmetric transmission^18^. Strong THz fields in metamaterials can lead to promising nonlinear and quantum responses for ultrafast, nonlinear THz photonics and plasmonics^19^,^20^,^21^,^22^. Remarkably, dynamic THz responses of graphene, liquid crystal, GeSbTe (GST), vanadium dioxide (VO_2_) and semiconductors enable active THz metamaterials that are excited by external stimuli via electric bias^23^,^24^,^25^,^26^,^27^, temperature^28^,^29^,^30^,^31^,^32^, photo excitation^33^,^34^,^35^,^36^,^37^,^38^ or MEMS^39^,^40^, which are capable of dynamic and flexible modulation of THz waves. Although chiral metamaterials were widely studied^5^,^6^,^7^,^8^,^9^,^10^,^11^,^12^,^16^,^17^,^18^, there are only a few attempts to realize dynamic modulation of the optical activity in the THz frequency^35^,^36^,^37^,^38^. The THz chiral metamaterials integrated with photoactive inclusions can accomplish tunable optical activity^35^,^36^, chirality switching^37^ and tunable linear polarization conversion^38^, which are controlled by external light illumination. At present, thermal-controlled chirality has been seldom reported^32^,^41^. The mid-infrared chiral metamaterials have been demonstrated to show the reversal of the circular dichroism sign and ultrafast tuning of pronounced circular conversion dichroism, respectively, enabled by temperature-controlled phase change materials Ge_3_Sb_2_Te_6_^32^ and Ge_2_Sb_2_Te_5_^41^. As one of the most important phase change materials, VO_2_ can exhibit an insulator-to-metal phase transition that can be electrically^27^, thermally^28^,^29^,^30^,^31^,^42^,^43^,^44^,^45^, or optically^46^ tuned. Generally, the temperature controlled phase transition of VO_2_ is much more preferable^28^,^29^,^30^,^31^,^42^,^43^,^44^,^45^. It is interestingly found that the VO_2_ transition behavior could be tailored by extreme nanogap^42^,^43^,^44^. The hysteresis curve can be narrowed and shift to a low temperature. Hybridizing VO_2_ with a metamaterial has been shown a large temperature activated tuning of the transmission without polarization conversion in the THz range. It is possible and worthwhile that the asymmetric transmission of linearly polarized wave and circular dichroism can be efficiently modulated by the phase transition effect of VO_2_, which provides an alternative route to realize switchable and functional THz devices. Actually, some other efficient approaches have been also presented to manipulate the polarization state with metasurfaces. For instance, broadband anomalous deflection can be observed with phase discontinuities^47^,^48^. Starting from the fundamental amplitude and phase, the polarization state of light can be tuned by controlling the time retardation with L-shaped microstructured surface^49^. However, they are not involved in the dynamic and flexible manipulation of the polarization state. A strategy will be demonstrated to realize a dynamic control of polarization conversion and asymmetric transmission in our scheme.

In this work, we report a novel metamaterial-based thermal switch to control artificial chirality and THz wave propagation exploiting the mechanism of VO_2_ phase transition. The hybrid THz metamaterial is comprised of an array of 90°-twisted E-shaped resonators with incorporated VO_2_ films. The orthogonal arrangement leads to a strong asymmetric transmission and circular dichroism in the bilayered metamaterial. The insulator-to-metal transition in VO_2_ films changes the resonator structure as well as the resonant frequency. The hybrid chiral metamaterial allows us to dynamically modify its chiral properties by controlling the temperature-dependent conductivity of VO_2_. Therefore, the thermal switching of the asymmetric transmission and circular dichroism can be implemented. Such dynamic control of phase transition metamaterials is of importance to acquire a variety of functionalities in the THz regime, such as filters, modulators and switches.

## Results

### Metamaterial design and simulation method

**Figure 1:**
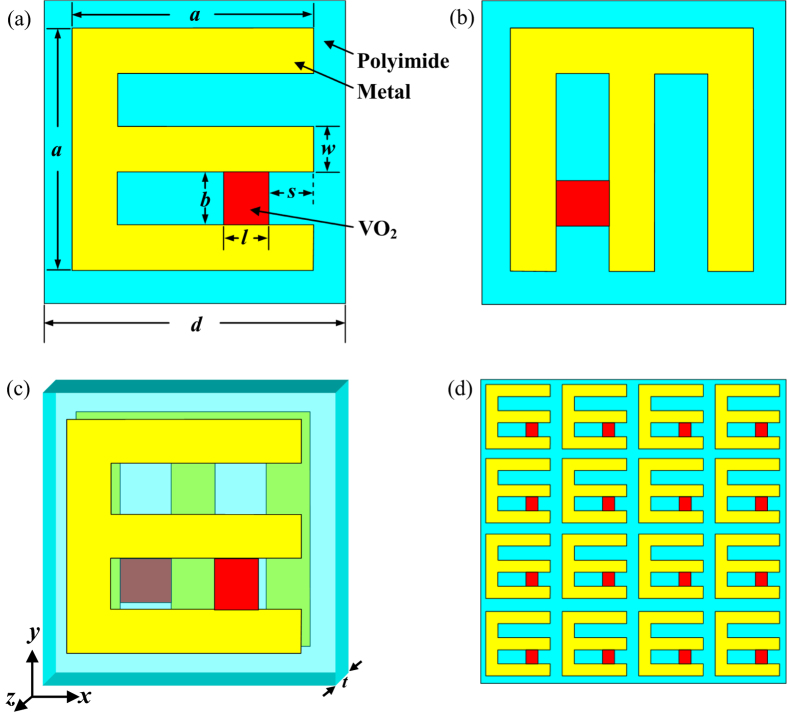
Schematic of the hybrid chiral metamaterial. (**a**) The front layer. (**b**) The back layer. (**c**) A unit cell in chiral metamaterial. (**d**) The top view of the metamaterial.

The proposed metamaterial configuration is sketched in Fig. 1. This hybrid metamaterial can be regarded as an array of square stereo E-shaped dimers embedded with VO_2_ inclusions. The period of the unit cell is *d* = 100 μm. Each dimer is composed of two spatially separated E-shaped gold resonators with a thickness of *t*_m_ = 200 nm. The two E-shaped resonators are geometrically identical, but the back layer is twisted by *θ* = 90° along the *z* axis with respect to the front one. The spacer dielectric layer is polyimide with a thickness of *t* = 16 μm. The E shape is square with a length of *a* = 80 μm. The width of the metallic strip is *w* = 15 μm. In order to realize a tunable THz metamaterial, VO_2_ films with a size of *b* = 17.5 μm and *l* = 15 μm are incorporated into one of the gaps of E-shaped resonators. The thickness of VO_2_ films is identical to that of metal layers, i.e., 200nm. The displacement of VO_2_ is described by *s*. VO_2_ film layer can be deposited on the polyimide dielectric by the reactive magnetron sputtering technique^28^, while VO_2_ islands and E-shaped metallic resonators can be fabricated by CF_4_/O_2_ plasma etching and the photolithography technique^28^,^30^. Under an external thermal excitation, VO_2_ film integrated into the chiral metamaterial will undergo an insulator-metal phase transition process, accompanied by conductivity change up to several orders of magnitude^30^. Therefore, the associated cross-polarization transmission properties can be modulated in this way. Compared with previous designs such as multilayered rosettes, swastikas and helix, the proposed 90°-twisted E-shaped resonators with incorporated VO_2_ films has relatively simple geometry, easy integration and more flexibly switching function for the cross-polarization transmission. Besides, a flat cross-polarization transmission and asymmetric transmission can be easily achieved with respect to other simple metamaterial structures. In addition, switching functionality based on phase transition property of VO_2_ can be applied to other metamaterial designs in microwave, THz and near-infrared frequencies^28^,^29^,^30^,^50^,^51^.

For a linearly polarized wave beam normally incident on the structure along the -*z* direction, Jones matrix is used to explore the polarization properties of the hybrid metamaterial. Jones matrix links the complex amplitudes of the incident to the transmitted field:


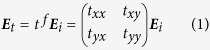


where the vectors ***E***_*i*_ and ***E***_*t*_ denote the incident and transmitted electric field, the matrix *t*^*f*^ represents the transmission matrix along forward propagation. The matrix *t*^*f*^ consists of 2 × 2 elements, in which *t*_*jk*_ and |*t*_*jk*_| indicate the transmission complex coefficients and amplitudes. The subscripts *j* and *k* correspond to the polarization states of the transmitted and the incident waves, which could be either *x* or *y* linear polarization. Here, the two layers are structurally identical, while the second layer structure is twisted clockwise by 90° around the *z* axis. So, *t*_xx_ is equal to *t*_yy_. Since the reciprocal theorem is applied, the transmission matrix *t*^*b*^ for backward propagation (along +*z* direction) can be derived as^9^,^52^:


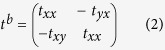


In order to further quantify the polarization properties of the metamaterial, the circular transmission coefficients can be inferred from the linearly polarized transmission matrix according to the following equation:


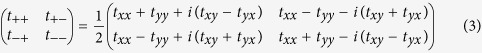


where “+” and “−” denote the right-handed (RCP) and left-handed circularly polarized waves (LCP), respectively.

Numerical simulations were performed in the THz range by means of the commercial software CST Microwave Studio, where the lossy polyimide spacer was assumed to have a relative permittivity *ε*_*p*_ = 2.4 + 0.005i 53 and gold was taken as a lossy metal with the conductivity of σ_Au_ = 4.561 × 10^7^ S/m. Generally, the complex dielectric properties of VO_2_ films in the THz range can be described by the Bruggeman effective-medium theory (EMT)^54^,


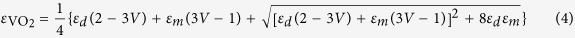


where *ε*_*d*_ and *ε*_*m*_, respectively, are dielectric constants of the insulating and metallic phase VO_2_ films, and *V* is the volume fraction of the metallic regions. For simplicity, the temperature-dependent conductivity 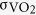 can alternatively qualify dynamic insulator-to-metal phase transition of VO_2_ films^28^,^29^,^30^. The relative permittivity of VO_2_ films in the insulating state is about 9, while the conductivity in the insulating state is smaller than 200 S/m and as high as an order of 10^5^ S/m in the metallic state^55^.

### Thermal switching properties of 90°-twisted E-shaped metamaterials embedded with VO_2_ films

VO_2_ films embedded into the bilayered E-shaped chiral metamaterial experience an insulator-to-metal phase transition under thermal excitation, further modulate the polarization conversion and asymmetric transmission due to the structure change of resonant elements. The electromagnetic responses of the hybrid metamaterials with VO_2_ films at different temperatures were modeled using the conductivity 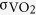. The change of VO_2_ conductivity from 200 to 10^5^ S/m corresponds to the insulator-to-metal phase transition^28^,^29^,^30^,^55^. The phase transition occurs on a picosecond timescale, as the VO_2_ conductivity reaches the value of the metallic state^29^. The thermal control of the THz metamaterial can be implemented by an attached thin Peltier heater/cooler^30^.

**Figure 2:**
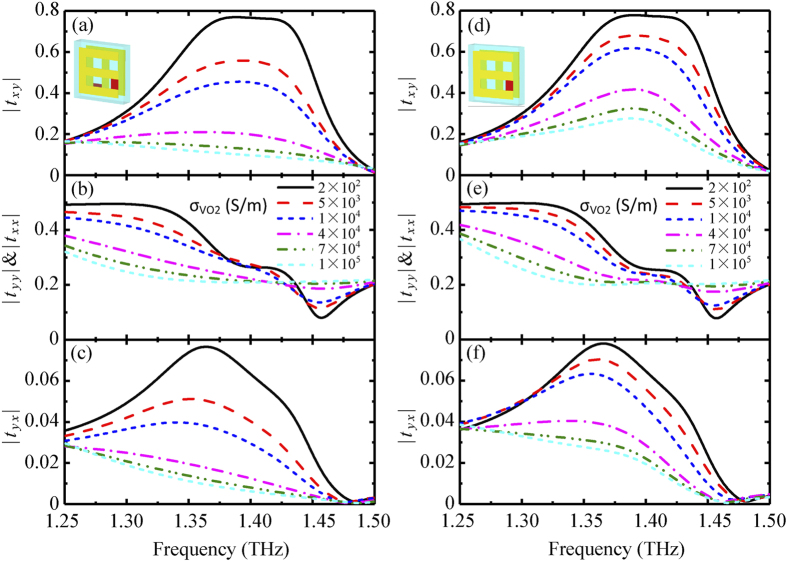
Simulated thermal switching properties of hybrid chiral metamaterials with *s* = 0 in forward propagation direction. (**a**–**c**) Cross-polarization and co-polarization transmission of hybrid chiral metamaterial incorporated by bilayered VO_2_ films in either layer as a function of VO_2_ conductivity. (**d**–**f**) Cross-polarization and co-polarization transmission of hybrid chiral metamaterial incorporated by single-layer VO_2_ film in the front layer as a function of VO_2_ conductivity. Insets indicate the hybrid metamaterials with bilayered and single-layer VO_2_ films.

Figures 2(a–c) show the simulated temperature dependence of transmission properties in the hybrid metamaterial incorporated by bilayered VO_2_ films for forwardly propagating electromagnetic waves along −*z* direction, where the displacement *s* = 0. In Fig. 2(a), when the VO_2_ film is in the insulating state with 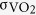  = 200 S/m, corresponding to the temperature of about 25 °C, the E-shaped metamaterial reveals flat y-to-x cross-polarization transmission spectrum in the region centered at 1.4 THz. The transmission maximum is close to |*t*_*xy*_| = 77%. Interestingly, this dispersion-free property of the cross-polarization transmitted light only occurs at a specific dielectric layer thickness. The dispersion-free property can be well engineered by the metamaterial’s parameters^56^,^57^,^58^. The intrinsic dispersion of the E-shaped metallic structure with embedded VO_2_ could actually be cancelled out by the thickness–dependent dispersion of the dielectric layer^56^. Hence, the cross-polarization transmission has dispersion free within a certain frequency range by properly choosing the thickness of the dielectric layer. As the temperature progressively increases, VO_2_ film undergoes a change from the insulator phase to the metal phase accompanied by an increasing conductivity. Accordingly, the y-to-x cross-polarization transmission has an obvious reduce. The complete insulator-to-metal phase transition of the metamaterial makes the y-to-x cross-polarization conversion into the “OFF” state. When 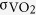 reaches 10^5^ S/m, corresponding to the temperature of about 85 °C, the y-to-x cross-polarization conversion |*t*_*xy*_| almost decreases less than 0.1 at about 1.4 THz. Particularly, the modulation depth of the y-to-x cross-polarization transmittance is as high as MOD = 96.6%, calculated by 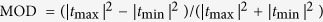.

On the contrary, no pronounced switching effect occurs for three other transmission coefficients. The co-polarization transmission *t*_*yy*_ and *t*_*xx*_depend weakly on 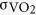 and are only thermally modulated in the range of 0.2–0.3 in Fig. 2(b). In Fig. 2(c), the other cross-polarization transmission *t*_*yx*_ is totally suppressed below 0.1 in the frequency range of 1.25–1.50 THz no matter what the conductivity 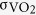 is. Considering the sample fabrication easiness, the transmission spectra of hybrid chiral metamaterial incorporated by single-layer VO_2_ film in the front layer are shown in Fig. 2(d–f). Similarly, an obvious thermal switching effect in single-layer VO_2_ film integrated metamaterial is available. However, the modulation depth of the y-to-x cross-polarization transmittance decreases to 76% due to slightly high transmission in the metallic state of VO_2_ film, here the metamaterial can be regards as a combination of E-shaped and 6-shaped resonators. Therefore, the hybrid chiral metamaterial incorporated with single-layer VO_2_ film can alternatively realize a thermal switching effect of the cross-polarization transmission.

Next, we mainly focus on the chiral switching response of the hybrid metamaterial with bilayered VO_2_ films. To visualize the thermal controlled polarization effect, the polarization rotation angle *θ* and its ellipticity angle *η* of the transmitted wave are calculated according to Eq. 5.


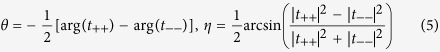


**Figure 3:**
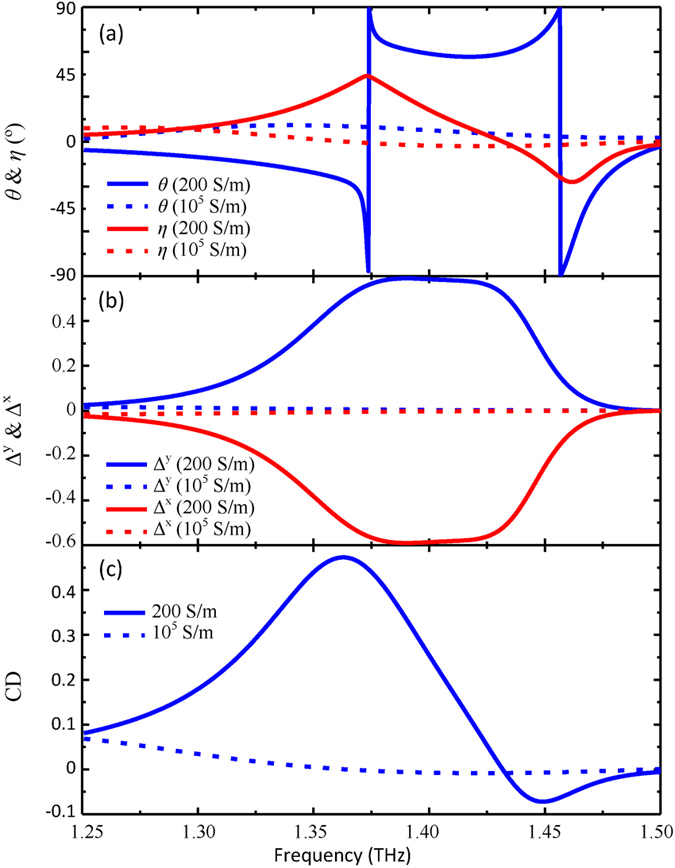
Simulated thermally controlled chirality in the hybrid metamaterial with bilayered VO_2_ inclusions for *s* = 0. (**a**) Polarization rotation azimuth angle *θ* and ellipticity *η*. (**b**) Asymmetric transmission parameter Δ. (**c**) Circular dichroism. The electric field of RCP light rotates anti-clockwise when looking into the coming beam.

Figure 3(a) clearly shows that such hybrid metamaterial is capable of effectively modulating both the polarization rotation angle *θ* and its ellipticity angle *η*. When VO_2_ film is insulating, at about 1.42 THz the transmitted wave is linearly polarized since the polarization rotation angle nearly approaches 60° and the ellipticity angle is close to 0, while at about 1.37 THz the transmitted wave is circularly polarized due to *η* = 45°. But VO_2_ film is metallic with its conductivity 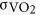 = 10^5^ S/m, both the polarization rotation angle *θ* and its ellipticity angle *η* are almost 0, thus its chirality is thermally switched off. The giant chirality modification results from the change of the effective metallic geometry when VO_2_ film experiences a phase transition. Importantly, the hybrid metamaterial can realize thermally controlled linear-to-circular polarization conversion at around 1.37 THz.

Since |*t*_*xy*_| ≠ |*t*_*yx*_| and |*t*_*xx*_| = |*t*_*yy*_|, the hybrid metamaterial will reveal the asymmetric transmission of linearly polarized wave. Asymmetric transmission for the linearly polarized wave refers to total intensity difference between two opposite directions and the associated asymmetric transmission parameter can be expressed as:


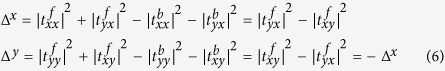


The insulator-to-metal phase transition of VO_2_ films enables this hybrid metamaterial to efficiently switch the asymmetric transmission of linearly polarized waves as shown in Fig. 3(b). Δ^*x*^ and Δ^*y*^ are exactly contrary to each other. Remarkably, the asymmetric transmission parameter can be thermally modulated between 0.6 and 0 in the frequency band of 50 GHz centered at 1.40 THz. Generally, the metamaterial exhibiting asymmetric transmission of linearly polarized wave is anisotropic and 3D chiral. Thus, the hybrid VO_2_ metamaterial can also thermally control its circular dichroism. The circular dichroism refers to differential transmittance between RCP and LCP waves, which can be calculated by CD = |*t*_++_|^2^ − |*t*_−−_|^2^. Interestingly, the circular dichroism strongly depends on the conductivity of the VO_2_ film shown in Fig. 3(c). The insulator-to-metal phase transition of the VO_2_ film leads to a complete switching effect of the circular dichroism with a maximum change of 0.45 at around 1.36 THz. Our demonstration could be of importance to modulate THz wave propagation and recognize interaction between the external stimuli and matter by detecting its chiral properties. The scheme could be exploited for developing novel highly efficient THz polarization modulators and thermal sensors.

**Figure 4:**
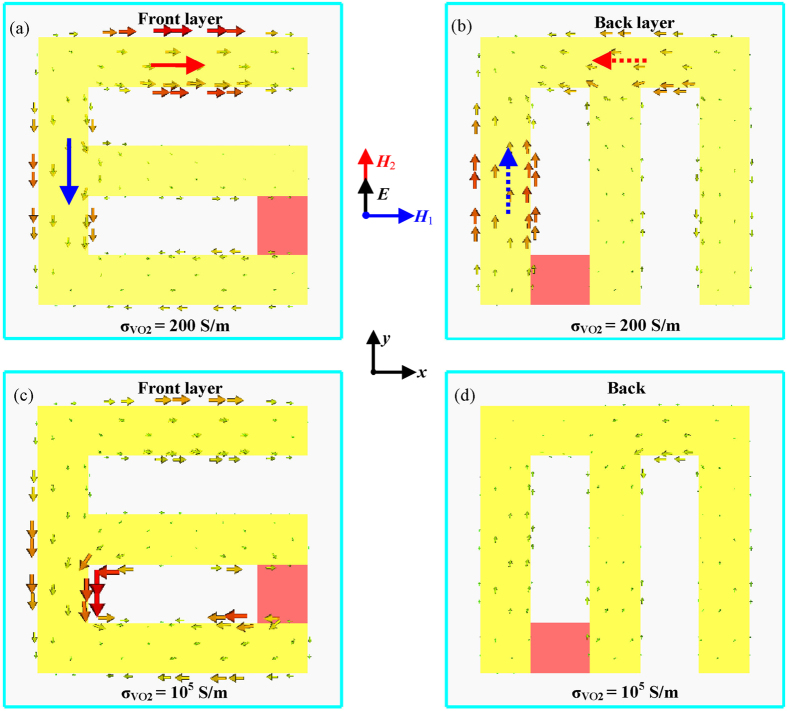
Distributions of induced surface currents of the bilayered hybrid VO_2_ metamaterial for y polarized incident wave at 1.4 THz. (**a**,**b**) Surface currents of the front and back layers without thermal excitation. (i.e., corresponding to 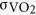 = 200 S/m). (**c**,**d**) Surface currents of the front and back layers with thermal excitation (i.e., corresponding to 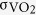 = 10^5^ S/m). The bold solid and dashed arrows indicate instantaneous directions of the current flow.

To clarify the physical origin of thermal-controlled switching polarization effect, the Born-Kuhn model can be recalled, in which two charged oscillators couple with each other^2^,^7^. The distributions of the instantaneous induced surface currents in the bilayered hybrid VO_2_ metamaterial at 1.4 THz are simulated and presented in Fig. 4. Without thermal excitation, VO_2_ film has a very low conductivity and the resonator is E-shaped. The strong antiphase current pairs excited by incident y-polarized wave can result in a magnetic response between the two layers shown in Fig. 4(a,b). The cross coupling happens between the induced magnetic field *H*_2_ and the incident electric field *E*. Therefore, the strong chirality can be observed. While with thermal excitation, VO_2_ film behaves as metal material and the resonator is 6-shaped. In this case, no strong antiphase current pairs and magnetic response are excited in Fig. 4(c,d). Thus, no strong chirality can be observed. It can be well understood that the thermal switching effect occurs due to the phase change of VO_2_.

**Figure 5:**
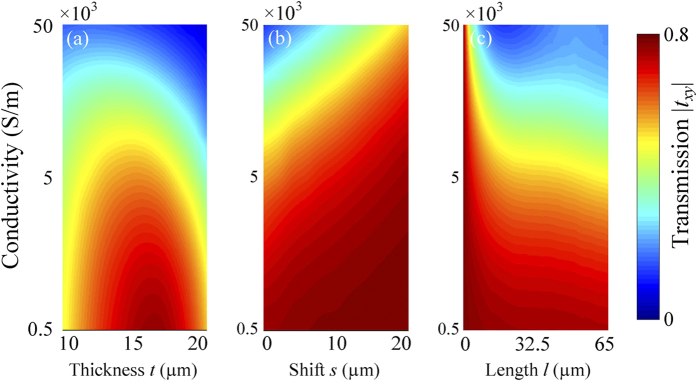
Geometrical dependence of the thermal switching phenomenon at the resonant frequency 1.4 THz by varying (**a**) the thickness of polyimide dielectric layer, (**b**) the shift of VO_2_ film and (**c**) the length of VO_2_ film.

In order to know how geometrical parameters of the metamaterial affect the thermal switching effect, we also perform numerical simulations of the hybrid metamaterial, in which the only one parameter is variable and the others are kept unchanged. The dependence of the switching properties at resonant frequency 1.4 THz is individually investigated on the thickness of polyimide dielectric layer, the displacement and length of VO_2_ film, shown in Fig. 5(a–c). When the thickness of polyimide dielectric layer increase from 10 to 20 μm, the hybrid metamaterial has an obvious thermal effect since the transmission strongly depends on the VO_2_ conductivity. In addition, the hybrid metamaterial has the largest switching modulation depth as the thickness of polyimide is about 17 μm. When the displacement *s* is less than 10 μm and the length *l* of VO_2_ films is larger than 10um, the thermal switching effect can be observed in Fig. 5(b,c). Particularly, the thermal switching effect can be engineered to work at other frequencies by changing geometrical parameters.

**Figure 6:**
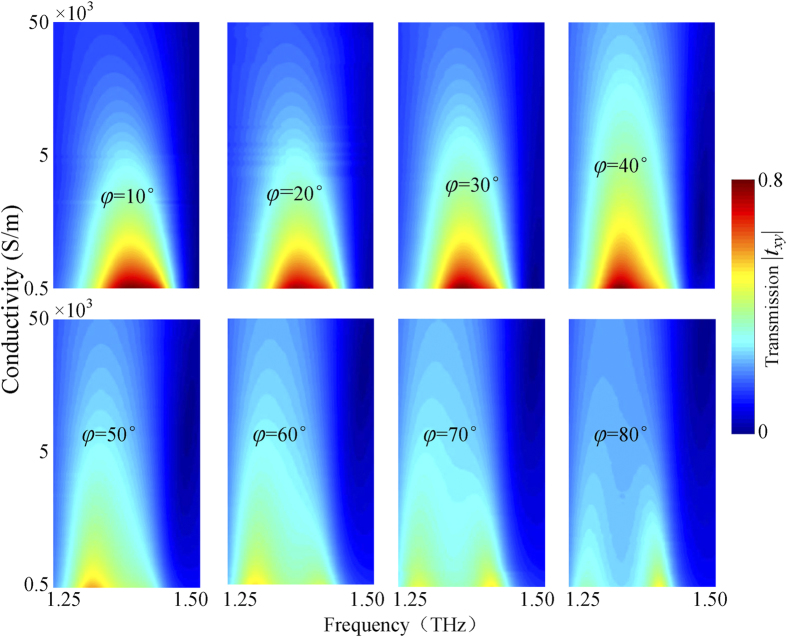
Angular dependence of the thermal switching effect in the bilayered hybrid metamaterial as a function of incident angle φ.

In general, extrinsic 2D and 3D chirality in intrinsically non-chiral metamaterials can be dominated and enhanced by increasing angles of incidence^6^,^11^. However, the oblique incidence usually weakens chiral properties in intrinsically chiral metamaterials. Therefore, it is necessary to investigate acceptable angle range for achieving such thermal switching effect. The angular dependence of the thermal switching effect is shown in Fig. 6. When the angle of incidence increases from 0° to 40°, the modulation depth of the cross-polarization transmission *t*_*xy*_ is almost kept unchanged, but the resonant frequency shifts slightly to red. For angles ranging from 50° to 80°, the modulation depth of the cross-polarization transmission *t*_*xy*_ has a rapid damping. As a result, no thermal switching effect can be observed in the hybrid chiral metamaterial at a large angle of incidence. It is worth mentioning that the thermal switching functionality in our metamaterial remains fairly available for a wide range of angles up to 40° and thus our design is robust and flexible to manipulate THz wave propagation.

## Discussion

In conclusion, we have demonstrated a thermal-controlled chiral switching in a hybrid THz metamaterial and the modulation depth of 96.6% is observed at about 1.4 THz for the linear cross-polarization transmittance. The insulator-to-metal phase transition of VO_2_ films embedded in two 90°-twisted E-shaped resonators promises a dynamic control on polarization rotation angle, circular dichroism and asymmetric transmission of linearly polarized light under external thermal excitation. The transmission maximum is close to |*t*_*xy*_| = 77% at about 1.4 THz. Particularly, our simulations show that the thermal switching functionality remains fairly available for a wide range of angles up to 40°. In addition, thermal switching effect can be engineered to work at other frequencies by changing geometrical parameters. Most importantly, the phase change material VO_2_ can be easily completed. Our work offers flexible polarization modulation in the THz frequency that would be highly beneficial for potential applications such as thermal switching and polarization sensitive detection.

## Methods

Numerical simulations were performed in the THz range by means of the commercial software CST Microwave Studio. In the model, the hybrid metamaterial is freestanding that could be possibly fabricated^15^. The boundary conditions in the x and y directions is set to unit cell boundary conditions due to its periodic structure. The lossy polyimide spacer was assumed to have a relative permittivity *ε*_*p*_ = 2.4 + 0.005i^53^ and gold can be taken as a lossy metal with the conductivity of σ_Au_ = 4.561 × 10^7^ S/m. Generally, the complex dielectric properties of VO_2_ films in the THz range can be described by the Bruggeman effective-medium theory (EMT)^54^. For simplicity, the temperature-dependent conductivity 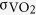 can alternatively qualify dynamic insulator-to-metal phase transition of VO_2_ films^28^,^29^,^30^. The relative permittivity of VO_2_ films in the insulating state is about 9, while the conductivity in the insulating state is smaller than 200 S/m and as high as an order of 10^5^ S/m in the metallic state^55^.

## Additional Information

**How to cite this article**: Lv, T. T. *et al.* Hybrid metamaterial switching for manipulating chirality based on VO_2_ phase transition. *Sci. Rep.* **6**, 23186; doi: 10.1038/srep23186 (2016).
